# Correction to: Minimally invasive fenestration for congenital hepatic cyst in infant

**DOI:** 10.1093/omcr/omac125

**Published:** 2022-10-22

This is a correction to: Yuki Muta, Akio Odaka, Seiichiro Inoue, Yuta Takeuchi, Yoshifumi Beck, Minimally invasive fenestration for congenital hepatic cyst in infant, *Oxford Medical Case Reports*, Volume 2022, Issue 7, July 2022, omac072, https://doi.org/10.1093/omcr/omac072

In the originally published version of this manuscript, Yuta Takeuchi's name was mis-spelled as Yuta Takechi.

This error has been corrected.

